# Implementation strategy for introducing a clinical skills examination to the Korean Oriental Medicine Licensing Examination: a mixed-method modified Delphi study

**DOI:** 10.3352/jeehp.2023.20.23

**Published:** 2023-07-17

**Authors:** Chan-Young Kwon, Sanghoon Lee, Min Hwangbo, Chungsik Cho, Sangwoo Shin, Dong-Hyeon Kim, Aram Jeong, Hye-Yoon Lee

**Affiliations:** 1 Department of Oriental Neuropsychiatry, College of Korean Medicine, Dongeui University, Busan, Korea; 2 Department of Medical Education, College of Korean Medicine, Kyung Hee University, Seoul, Korea; 3 Department of Korean Medicine Ophthalmology & Otolaryngology & Dermatology, Daegu Hanny University, Gyeongsan, Korea; 4 Department of Korean Internal Medicine, Seoul Korean Medicine Hospital of Daejeon University, Seoul, Korea; 5 Division of Applied Medicine, School of Korean Medicine, Pusan National University, Yangsan, Korea; 6 Department of Internal Medicine, College of Korean Medicine, Daejeon University, Daejeon, Korea; 7 Department of Pediatrics, College of Korean Medicine, Gachon University, Seongnam, Korea; 8 Division of Humanities and Social Medicine, School of Korean Medicine, Pusan National University, Yangsan, Korea; Hallym University, Korea

**Keywords:** East Asian traditional medicine, Oriental medicine, Education, Clinical competence, Licensure

## Abstract

**Purpose:**

This study investigated the validity of introducing a clinical skills examination (CSE) to the Korean Oriental Medicine Licensing Examination through a mixed-method modified Delphi study.

**Methods:**

A 3-round Delphi study was conducted between September and November 2022. The expert panel comprised 21 oriental medicine education experts who were officially recommended by relevant institutions and organizations. The questionnaires included potential content for the CSE and a detailed implementation strategy. Subcommittees were formed to discuss concerns around the introduction of the CSE, which were collected as open-ended questions. In this study, a 66.7% or greater agreement rate was defined as achieving a consensus.

**Results:**

The expert panel’s evaluation of the proposed clinical presentations and basic clinical skills suggested their priorities. Of the 10 items investigated for building a detailed implementation strategy for the introduction of the CSE to the Korean Oriental Medicine Licensing Examination, a consensus was achieved on 9. However, the agreement rate on the timing of the introduction of the CSE was low. Concerns around 4 clinical topics were discussed in the subcommittees, and potential solutions were proposed.

**Conclusion:**

This study offers preliminary data and raises some concerns that can be used as a reference while discussing the introduction of the CSE to the Korean Oriental Medicine Licensing Examination.

## Graphical abstract

[Fig f2-jeehp-20-23]

## Introduction

### Background

The core components of medical education are knowledge, attitudes, and skills. Accordingly, the importance of practical evaluation in medical education has been emphasized [1]. To evaluate the clinical competence of medical students, some objective evaluation tools, such as the clinical performance examination (CPX) and objective structured clinical examination (OSCE), have been used [2]. The CPX evaluates whether history-taking, physical examination, and patient education have been performed correctly and whether desired patient–physician interactions have been achieved in the course of interviewing a standardized patient (SP) [3]. In the OSCE, the performance appropriateness of basic clinical procedures is evaluated based on an SP or via a model [3]. In South Korea, a clinical skills examination (CSE) has been implemented since 2010 as part of the Korean Medical Licensing Examination [4]. The CSE was introduced for the first time for Korean Dental Licensing Examinations in Korea in 2021 [5]. During the last decade, the practical ability of oriental medicine doctors has been emphasized [6]. As oriental medicine includes various unique medical techniques such as pulse examination and acupuncture, an emphasis has been placed on strengthening the practice-based competence of oriental medicine doctors [6]. According to a recent scoping review, there were 25 studies on the OSCE and CPX vis-à-vis oriental medicine in South Korea between 2011 and 2022, and this number had increased significantly over the last 3 years (i.e., studies from 2020 to 2022 accounted for 52% of the total) [7].

The Delphi method is used when there is no existing basis in relation to a statistical model, knowledge in the field in question is incomplete, and expert judgment is considered important [8]. It has been used widely in the health science and medical education fields [9]. Notably, oriental medicine doctors are very similar to traditional Chinese medicine doctors in China and Taiwan, although they differ in their knowledge systems and level of medical practice guaranteed by their license. Thus, there is no empirical evidence for reference in the field of studies on the Korean Oriental Medicine Licensing Examination. Therefore, the validity of introducing the CSE to the Korean Oriental Medicine Licensing Examination, which requires investigation, may be useful to address this gap in the literature.

### Objectives

The purpose of this study was to develop implementation strategies for the CSE for the Korean Oriental Medicine Licensing Examination. The detailed objectives included the following 3 questions: (1) What are the appropriate clinical presentations for the CSE? (2) What are the appropriate basic clinical skills for the CSE? (3) When and how should the CSE be introduced?

## Methods

### Ethics statement

This study complied with the Declaration of Helsinki. It was approved by the Institutional Review Board of Gachon University Gil Korean Medical Hospital (GIRB-22-108) on September 5, 2022. Informed consent was obtained from all the study participants.

### Setting

This Delphi study was completed electronically between July and November 2022.

### Study design

A 3-round Delphi study following the recommended guidelines for the conducting and reporting of Delphi studies was undertaken [10].

### Setting of the problem area

To set the problem area, the authors conducted 4 investigations for clarification: a comprehensive literature review, an email-based survey of oriental medicine doctors registered with the Association of Korean Medicine (response rate: 9.28%; 2,221/23,946) and of professors at 12 oriental medicine schools (response rate: 41.4%; 206/500), and an advisory board meeting with the participation of the Association of Korean Medicine Colleges. The research methods and results for setting the problem area are described in Supplement 1.

### Respondent group

The expert panel was set up based on official recommendations from related official organizations, including oriental medicine universities and the official subdivision of the oriental medicine society. A panel of 21 experts was formed, including 12 oriental medicine education experts and 9 subdivision representatives.

### Working group

The working group comprised 8 researchers. All the working group members were oriental medicine doctors, and 7 of them were professors at oriental medicine schools. The principal researcher (H.Y.L.) has a doctoral degree in medical education.

### Instrumentation: 3-round web-based Delphi study

The Delphi method was implemented over 3 rounds. Each round was conducted as an email-based survey to a panel of experts. Anonymity was guaranteed, and controlled feedback was provided. The objectives of the first, second, and third rounds were distinguished. In the first round (September 15–22, 2022), opinions were gathered from the expert panel on the list of potential candidates for the CPX and OSCE. The validity of introducing each item into the Korean Oriental Medicine Licensing Examination was measured with 6 (for basic clinical skills) and 7 (for clinical presentations) questions, including “necessity,” “feasibility,” and the RUMBA mnemonic (i.e., “relevant,” “understandable,” “measurable,” “behavioral,” and “achievable”). The questions were to be answered on a 3- or 5-point Likert scale. As there is no agreed mode of application of the Likert scale for this question, the use of 3 or 5 points was decided through consensus among the authors. Open-ended questions enabled the free expression of opinions vis-à-vis clinical presentations or basic clinical skills. Four subcommittees were formed by requesting official recommendations from related official organizations in connection to pulse examination, acupuncture, *chuna*, and *sasang* constitution to discuss the comments raised in the open-ended questions. These 4 topics are the hallmark clinical areas of oriental medicine in Korea. The subcommittees also discussed how to successfully implement these topics into the CSE for the Korean Oriental Medicine Licensing Examination. In the second (September 27–October 6, 2023) and third (October 22–November 3, 2023) rounds, the validity of detailed methods for the CSE was investigated. The third round included questions on which no consensus was reached in the second round. In this round, options other than the most frequent one according to the professor’s response could also be selected. The results of discussions in the subcommittees were provided, and the expert panel was asked to respond about whether or not it agreed with the aforesaid results.

### Protocol

**Fig. 1.**
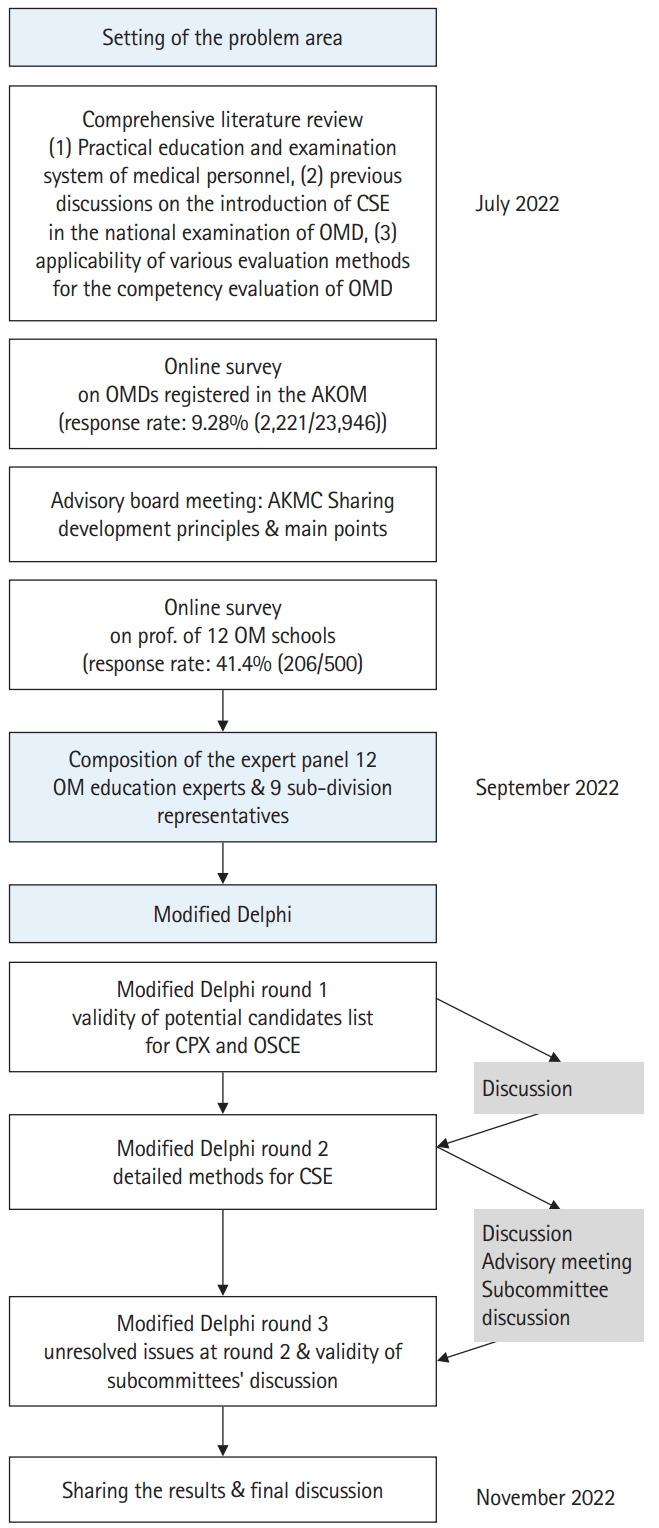
Process of the study, including the 3-round Delphi survey. CSE, clinical skills examination; OMD, oriental medicine doctor; AKOM, Association of Korean Medicine; AKMC, Association of Korean Medicine (oriental medicine) Colleges; OM, oriental medicine; CPX, clinical practice examination; OSCE, objective structured clinical examination.

The protocol of this study is presented in Supplement 2 and summarized in Fig. 1.

### Data analysis

The average scores of the responses to the clinical presentations of the CPX and the list of basic clinical skills in the first round were calculated and ranked. Descriptive statistics were used to assess whether a consensus was reached in the second and third rounds. An agreement rate of 66.7% or higher was adopted as the definition of consensus.

## Results

The clinical experience of the expert panel varied from 10 to 31 years (average, 19.3 years). The regional distribution was as follows: Seoul City/Incheon City/Gyeonggi-do (n=5); Busan City/Ulsan City/Gyeongsangnam-do (n=14); Gangwon-do (n=3); Gwangju City/Jeollanam-do (n=2); Daegu City/Gyeongsangbuk-do (n=2); Daejeon City/Chungcheongnam-do (n=2); Jeollabuk-do (n=2); and Chungcheongbuk-do (n=1).

### First round: Questions 1 and 2

The top 5 clinical presentations of the CPX included back pain (total average score=24.7), neck pain (24.4), omalgia (24.4), headache (24.2), and ankle pain (24.1) (Table 1). The top 5 basic clinical skills included cupping (total average score=19.3), moxibustion (19.0), acupuncture (19.0), medical records/medical certificate (18.9), and pharmacopuncture (18.9) (Table 2). The concerns raised from the open-ended questions during the first round were grouped into 4 clinical topics (Table 3).

### Second round: Question 3

A consensus was reached for 9 items in the second round, including items (b)–(j) (Table 4). In the second round, the 4 subcommittees discussed the concerns raised from the open-ended questions in the first round. The results of the discussion were summarized and presented in the third round. Items (d), (f), and (i) were included in the third round despite reaching a consensus, as some of the concerns raised warranted further discussion (Table 4).

### Third round: Question 3

No consensus was reached on item (a) in the second and third rounds (i.e., the year of introduction of the CSE). Consensus was reached for items (d), (f), and (i) in the second and third rounds. Among them, the consensus reached for items (d) and (f) differed from that in the second round: (d) the test time per station was 12 minutes (90.5%), and (f) the appropriate timing for the CSE was before the written test, and it should only be for a prospective graduate (66.7%). However, the same consensus was reached for item (i), namely, the CPX should be rated by 2 SPs (1 for acting and 1 for scoring) (71.4%) (Table 4). The feasibility evaluated through the subcommittees’ discussion on concerns raised was 3 points or higher on average for both relevant and measurable factors (Table 5). As this study was preliminary, the authors did not plan further rounds to arrive at a consensus.

## Discussion

### Interpretation

This Delphi study investigated 2 topics: a list of clinical presentations and basic clinical skills that could be used and/or should be developed if the CSE were to be introduced into the Korean Oriental Medicine Licensing Examination and a detailed strategy for its implementation. Although the number of OSCE or CPX studies focusing on oriental medicine doctors has recently increased, educational resources for the CSE for oriental medicine doctors still need to be developed [7]. This study listed potential candidates’ clinical presentations and basic clinical skills for the introduction of the CSE into the Korean Oriental Medicine Licensing Examination through a comprehensive literature review and a survey of 2,221 oriental medicine doctors. Each item was evaluated by an expert panel, and our findings suggest tasks to prioritize for introducing the CSE to the Korean Oriental Medicine Licensing Examination. The priority lists of clinical presentations and basic clinical skills included “back pain,” “neck pain,” “omalgia,” “headache,” and “ankle pain” for clinical presentations (Table 1), as well as “cupping,” “moxibustion,” “acupuncture,” “medical records/medical certificate,” and “pharmacopuncture” for basic clinical skills (Table 2). The expert panel reached a partial consensus on the detailed implementation strategy in the second and third rounds. Notably, however, the consensus rate was low for the timing of the introduction of the CSE. It may be important to address the survey items for which a consensus was not reached, including the development of CSE resources and the formulation of detailed implementation strategies, in future research. Moreover, to establish a comprehensive evaluation framework, it is imperative to promptly initiate formal development procedures.

### Limitations

This study has several limitations. First, there are limitations with regard to the expert panel setting. This Delphi study established an expert panel based on official recommendations from related official organizations in South Korea. However, this does not guarantee that the participants were highly recognized education experts on oriental medicine education, especially the CSE. Nevertheless, as the importance of clinical skills training in oriental medicine education has only been emphasized relatively recently [7], and there are no clear criteria for the selection of such experts, the authors had no choice but to establish the expert panel with this limitation. Second, the expert panel’s opinions were not weighted. The lists of clinical presentations and basic clinical skills, which constituted the main components of the questionnaire in this study, might have been imbalanced owing to the expertise of the respondents based on clinical division. However, this study did not acknowledge differences in clinical expertise and gave equal weight to the opinions of the experts on all clinical presentations and basic clinical skills lists. Third, there was some discontinuity between the second and third rounds. Therefore, the stability of the responses could not be evaluated, and the possibility of a coincidence cannot be excluded from the consensus obtained. Finally, a Likert scale was used to evaluate items on necessity, feasibility, and RUMBA was evaluated for the clinical presentations and basic clinical skills. The importance was listed in order by summing the responses. However, as the legitimacy and weighting of the summation of these scales have not been verified, it is acknowledged that the methodology of this study is somewhat arbitrary.

### Suggestions for further research

Further research in this field may refer, but need not be limited, to the results of this preliminary study. First, basic research to introduce the CSE into the Korean Oriental Medicine Licensing Examination should be carried out. Studies should focus on areas such as developing a CSE implementation guide and simulation to evaluate the likelihood of the successful implementation of the guide. If necessary, a feasibility study on the introduction of the simulator can be conducted to facilitate the introduction of the CSE. Following this, a list of items to be used in the CSE should be officially developed, and the method of evaluation for each item should be established. Second, research on education and evaluation for the introduction of the CSE should be carried out. Research can be conducted to set a passing mark if the CSE is introduced to the Korean Oriental Medicine Licensing Examination and to develop educational textbooks for assessors and SPs. Finally, it would be desirable to conduct 1–3 simulation CSEs before the official introduction, and related laws and regulations must be revised for the official introduction of the CSE to the Korean Oriental Medicine Licensing Examination.

### Conclusion

The main clinical presentations for the CSE were back pain, neck pain, omalgia, headache, and ankle pain. The essential basic clinical skills were cupping, moxibustion, acupuncture, medical records/medical certificate, and pharmacopuncture. A consensus on the implementation time of CSE into the Korean Oriental Medicine Licensing Examination was still not reached. This study is the first to propose the introduction of the CSE to the Korean Oriental Medicine Licensing Examination. It provides preliminary data that can be used as a reference while discussing the introduction of the CSE.

## Figures and Tables

**Figure f2-jeehp-20-23:**
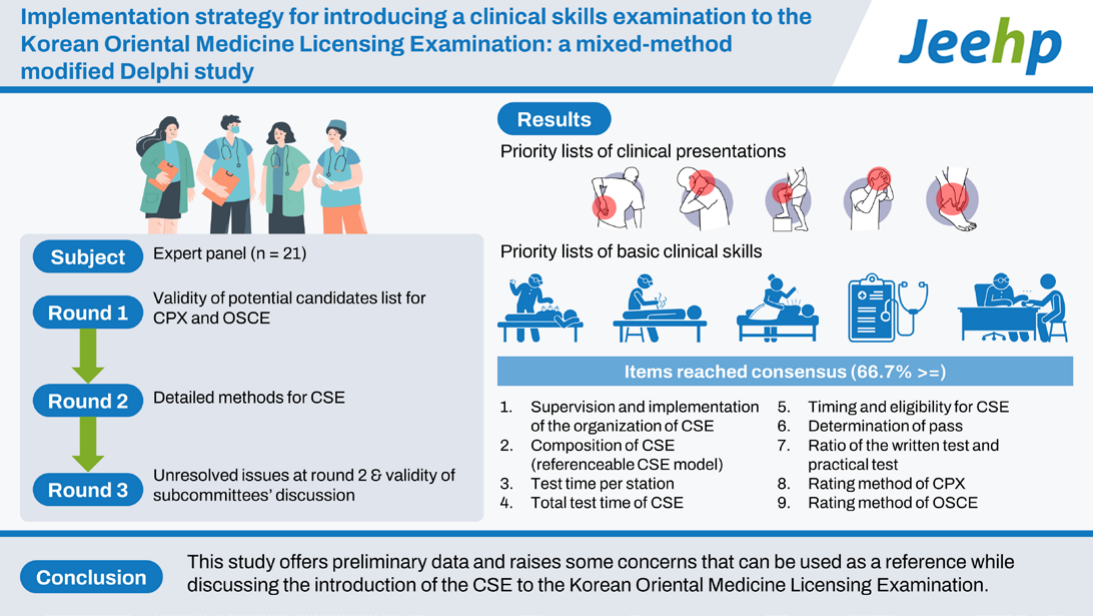


**Table 1.** Expert panel’s evaluation of the clinical presentations of clinical skills examination of the Korean Oriental Medicine Licensing Examination (first round, top 10 lists) (N=21)

Clinical presentations	Necessity (1 to 5)	Feasibility (1 to 5)	RUMBA (1 to 3)					Total score (response rate)
			Relevant	Understandable	Measurable	Behavioral	Achievable	
Backache	5.00	4.80	3.00	2.95	2.95	2.95	3.00	24.7 (95.24)
Neck pain	4.86	4.76	3.00	2.95	2.90	2.95	3.00	24.4 (100.00)
Omalgia	4.80	4.75	3.00	2.95	2.90	2.95	3.00	24.4 (95.24)
Headache	4.95	4.71	3.00	2.95	2.71	2.86	3.00	24.2 (100.00)
Ankle pain	4.65	4.60	2.95	2.95	2.95	2.95	3.00	24.1 (95.24)
Knee pain	4.70	4.65	3.00	2.90	2.90	2.85	2.95	24.0 (95.24)
Dizziness	4.76	4.62	2.95	2.90	2.81	2.81	2.90	23.8 (100.00)
Chronic abdominal pain/dyspepsia/heartburn	4.67	4.52	2.95	2.90	2.71	2.81	2.95	23.5 (100.00)
Arm pain	4.40	4.55	2.90	2.90	2.85	2.85	2.95	23.4 (95.24)
Dysmenorrhea	4.40	4.60	2.95	2.95	2.75	2.75	2.95	23.4 (95.24)

**Table 2.** Expert panel’s evaluation of basic clinical skills of the Korean Oriental Medicine Licensing Examination (first round, top 10 lists) (N=21)

Basic clinical skills	Necessity (1 to 5)	RUMBA (1 to 3)					Total score (response rate)
		Relevant	Understandable	Measurable	Behavioral	Achievable	
Cupping	4.81	2.95	2.95	2.81	2.86	2.95	19.3 (100.00)
Moxibustion	4.76	2.90	2.95	2.67	2.86	2.90	19.0 (100.00)
Acupuncture	4.81	2.86	2.95	2.57	2.86	2.95	19.0 (100.00)
Medical records/medical certificate	4.57	2.90	2.90	2.71	2.90	2.86	18.9 (100.00)
Pharmacopuncture	4.62	2.90	2.95	2.62	2.86	2.90	18.9 (100.00)
Cardiopulmonary resuscitation/defibrillation	4.48	2.71	2.95	2.86	2.90	2.81	18.7 (100.00)
Wound dressing	4.40	2.85	2.95	2.75	2.90	2.80	18.7 (95.24)
Obtaining consent	4.33	2.81	2.95	2.67	2.86	2.76	18.4 (100.00)
Electrocardiography	4.00	2.67	2.86	2.95	2.90	2.81	18.2 (100.00)
Abdominal examination	4.52	2.76	2.90	2.33	2.81	2.81	18.1 (100.00)

**Table 3.** Concerns about 4 clinical topics raised during the first round Delphi study for the Korean Oriental Medicine Licensing Examination

Topic	Concerns
Pulse examination	No standardized method for measuring the accuracy of pulse examination
Acupuncture	Scope of evaluation of these OM treatments for SPs, given the limited number of SPs, and safety and ethical issues
*Chuna*	Scope of evaluation of these OM treatments for SPs, given the limited number of SPs, and safety and ethical issues
*Sasang* constitution	Whether the diagnostic process of the *sasang *constitution should be evaluated, given the acceptable CPX time and its difficulty

OM, oriental medicine; SP, standardized patient; CPX, clinical practice examination.

**Table 4.** Delphi questionnaire, responses, and preliminary consensus on detailed methods for the CSE in the Korean Oriental Medicine Licensing Examination

Item	Options	Most frequent response of professors at the 12 OM schools (N=206)	Agreement rate on the most frequent response of the professors (second round) (N=21)	Agreement rate on the options (third round) (N=21)
(a) Year of introduction of CSE	(1) 2025, (2) 2026, (3) 2027, (4) 2028, (5) 2029, (6) Other	(1) 2025 (37.9%)	(1) 2025 (42.9%)	(1) 2025 (19.0%), (3) 2027 (47.6%), (5) 2029 (33.3%)
(b) Supervision and implementation of the organization of CSE	(1) KHPLEI, (2) KHPLEI and University, (3) regional autonomy under the responsibility of KHPLEI, (4) University	(1) KHPLEI (68.9%)	(1) KHPLEI (100%)	Not required, as a consensus was reached in the previous round.
(c) Composition of CSE (referenceable CSE model)	(1) 6 CPXs, 6 inter-station tests, and 6 single OSCEs (the CSE model of MDs in South Korea, 2009–2020); (2) 9 CPXs and 1 combined-OSCE of 3 skills (the CSE model of MDs in South Korea, 2021–current); (3) 3 result-assessments and 3 procedure-assessments (the CSE model of dentists in South Korea); (4) case analysis, 4 inter-student demonstrations (similar to OSCE), and 2 oral tests (the CSE model of TCM doctors in China); (5) 12 CPXs and 12 patient notes (the CSE model of MDs in US [i.e., USMLE]); (6) others	(2) 9 CPXs and 1 combined-OSCE of 3 skills (the CSE model of MD in South Korea, 2021–current) (38.3%)	(2) 9 CPXs and 1 combined-OSCE of 3 skills (the CSE model of MD in South Korea, 2021–current) (95.2%)	Not required, as a consensus was reached in the previous round.
(d) Test time per station^a)^	(1) 10 minutes, (2) 12 minutes, (3) 15 minutes, (4) other	(3) 15 minutes (38.3%)	(3) 15 minutes (85.7%)	(2) 12 minutes (90.5%), (3) 15 minutes (9.5%)
(e) Total test time of CSE	(1) 2 hours, (2) 3 hours, (3) 4 hours, (4) 5 hours, (5) 6 hours, (6) other	(2) 3 hours (52.4%)	(2) 3 hours (90.5%)	Not required, as a consensus was reached in the previous round.
(f) Timing and eligibility for CSE^a)^	(1) Before the written test, prospective graduate; (2) after the written test, prospective graduates; (3) after the written test, only prospective graduates who have passed the written test; (4) regardless of the written test and eligibility	(3) After the written test, only prospective graduates who have passed the written test (45.6%)	(3) After the written test, only prospective graduates who have passed the written test (85.7%)	(1) Before the written test, prospective graduates (66.7%); (2) after the written test, prospective graduates (14.3%); (3) after the written test, only prospective graduates who have passed the written test (14.3%)
(g) Determination of pass	(1) Passing both the written and clinical skills exams; (2) passing according to the combined score of the written test and clinical skills exams; (3) other	(1) Passing both the written and practical exams, respectively (71.4%)	(1) Passing both the written and practical exams, respectively (95.2%)	Not required, as a consensus was reached in the previous round.
(h) Ratio of the written test and practical test (in case of combining the scores of the written test and clinical skills exams)	(1) 8:2, (2) 7:3, (3) 6:4, (4) 5:5, (5) 4:6, (6) 3:7, (7) 2:8	(1) 8:2 (46.0%)	(1) 8:2 (76.2%)	This consensus was invalidated because the combination of the scores of the written test and clinical skills exams did not reach a consensus. (item [g])
(i) Rating HT, PE, and ED of CPX (PPI is scored by 1 SP who is acting)^a)^	(1) By 1 university teacher at schools of OM; (2) by 2 university teachers at schools of OM; (3) by 1 SP (acting and scoring); (4) by 2 SPs (1 for acting and the other 1 for scoring); (5) other	(4) By 2 SPs (1 for acting and the other 1 for scoring) (46.8%)	(4) By 2 SPs (1 for acting and the other 1 for scoring) (76.2%)	(1) By 1 university teacher at schools of OM (28.6%); (4) by 2 SPs (1 for acting and the other 1 for scoring) (71.4%)
(j) Rating OSCE	(1) By 1 university teacher at schools of OM (scoring); (2) by 2 university teachers at schools of OM; (3) other	(1) By 1 university teacher at schools of OM (66.1%)	(1) By 1 university teacher at schools of OM (90.5%)	Not required, as a consensus was reached in the previous round.

CSE, clinical skills examination; OMD, Oriental medicine doctor; OM, Oriental medicine; KHPLEI, Korea Health Personnel Licensing Examination Institute; CPX, clinical practice examination; OSCE, objective structured clinical examination; MD, medical doctor; TCM, traditional Chinese medicine; USMLE, United States Medical Licensing Examination; HT, history taking; PE, physical examination; ED, education; PPI, patient–physician interaction; SP, standardized patient.

a) An agreement was reached in the second round, but it was included in the third round because it required further discussion.

**Table 5.** Subcommittees’ discussion of concerns during the Delphi rounds for the Korean Oriental Medicine Licensing Examination (N=21)

Clinical topic	Results of discussion	Feasibility (1 to 5): (third round)	
		Relevant	Measurable
Pulse examination	Pulse examination can be included in the CPX, but only the procedure may be evaluated on an SP, and the results of the pulse examination are presented by card.	3.86	3.57
	It is realistic to evaluate the pulse examination in OSCE by evaluating the speed of the SP’s pulse.	3.52	3.52
Acupuncture	Acupuncture can be evaluated on an OSCE, but only the procedure may be evaluated, and actual needle insertion and manipulation should be performed on an artificial pad, not an SP.	4.57	4.57
*Chuna*	*Chuna* can be evaluated on an OSCE, but only some techniques that are less harmful to the SP should be used.	3.81	3.81
	ICT can be evaluated on an OSCE, procedures such as questions about contraindications may be evaluated with an SP, and actual electrode placement and device operation could be performed on mannequins.	4.29	4.14
*Sasang* constitution	*Sasang* constitution diagnosis should be provided in the test instructions.	3.48	3.29
	*Sasang* constitution practice can be included in clinical presentations of a CPX, but it is recommended to conduct a KCD diagnosis and *sasang* constitution practice in parallel for clinical expression.	3.57	3.57

CPX, clinical practice examination; SP, standardized patient; OSCE, objective structured clinical examination; ICT, interferential current therapy; KCD, Korean Standard Classification of Diseases.

## Supplementary materials

Supplement 1. Setting of the problem area.

Supplement 2. Protocol of the study.

Supplement 3. Audio recording of the abstract.
