# Supplementary material for: Implementation strategy for introducing a clinical skills examination to the Korean Oriental Medicine Licensing Examination: a mixed-method modified Delphi study
Source: J Educ Eval Health Prof. 2023 Jul 17;20:23. doi: 10.3352/jeehp.2023.20.23 (PMC10432826; doi:10.3352/jeehp.2023.20.23)
Supplement: Supplementary file 1 — Supplement 1. Setting of the problem area. [file jeehp-20-23-suppl1.docx]

**Supplement 1. Setting of the problem area**

*1-1. Comprehensive literature review*

During July 2022, through a comprehensive search in the database including MEDLINE via PubMed and Google Scholar, a literature search was conducted on the practical education and examination system of domestic and foreign medical personnel. Domestic medical personnel surveyed included physician and dentist. The foreign medical personnel surveyed included physician and traditional Chinese medicine doctors in China, traditional Chinese medicine doctors in Taiwan, physician (i.e., USMLE), osteopathic physician (i.e., USMLE or COMLEX-USA), and acupuncturists (i.e., NCCAOM certification) in US. In addition, the results of previous discussions on the introduction of clinical skill examinations in the national examination of oriental medicine doctors were investigated, including the ‘Research on the Implementation Plan for Improving Subjects for the National Examination for Oriental Medicine Doctors’ conducted by Jong Hyeong Park with the support of the Korea Health Personnel Licensing Examination Institute in 2008. Lastly, the applicability of various evaluation methods for the competency evaluation of oriental medicine doctors was reviewed, based on the computer-based test such as XR-acupuncture platform.

*1-2. Email-based survey of oriental medicine doctors*

During August 2022, an email-based survey of oriental medicine doctors was conducted. They responded in favor of and against the necessity of the clinical skills examination into the Korean Oriental Medicine Licensing Examination. They also answered the possible timing of the introduction of the clinical skills examination and the competencies that should be assessed. Finally, they were introduced to potential clinical presentations and basic clinical skills candidates, and were asked to give importance to their inclusion in the clinical skills examination.

Table 1-2-1. Demographic data of the respondents (N = 2,221)

| Variables | | N | Share of total respondents (%) |
| --- | --- | --- | --- |
| Age (years) | 20s | 294 | 13.2 |
|  | 30s | 893 | 40.2 |
|  | 40s | 598 | 26.9 |
|  | 50s | 350 | 15.8 |
|  | ≥60s | 86 | 3.9 |
| Sex | Male | 1,555 | 70 |
|  | Female | 666 | 30 |
| Affiliation | University (basic department) | 28 | 1.3 |
|  | University (clinical department) | 59 | 2.7 |
|  | Oriental Medicine Hospital | 454 | 20.4 |
|  | Oriental Medicine Clinic | 1,372 | 61.8 |
|  | Public Medical Institution | 221 | 10 |
|  | Other (Research Institution) | 87 | 3.9 |
| Resident education | None | 1,528 | 68.8 |
|  | Completed Internship only | 143 | 6.4 |
|  | Complete Residency | 550 | 24.8 |
| License acquisition year | 2018–2022 | 640 | 28.8 |
|  | 2013–2017 | 474 | 21.3 |
|  | 2008–2012 | 335 | 15.1 |
|  | 2003–2007 | 278 | 12.5 |
|  | 1998–2002 | 238 | 10.7 |
|  | 1993–1997 | 110 | 5 |
|  | 1988–1992 | 91 | 4.1 |
|  | 1983–1987 | 33 | 1.5 |
|  | before 1982 | 22 | 1 |

Table 1-2-2. Summary of survey results (N = 2,221)

| Questions | Responses | n (Share of total respondents (%)) |
| --- | --- | --- |
| Necessity | Strongly positive | 1,060 (47.7) |
|  | Slightly positive | 759 (34.2) |
|  | Neutral | 239 (10.8) |
|  | Slightly negative | 82 (3.7) |
|  | Strongly negative | 81 (3.6) |
| Year of introduction of clinical skills examination | 2025 | 1,328 (64.5) |
|  | 2026 | 221 (10.7) |
|  | 2027 | 245 (11.9) |
|  | 2028 | 99 (4.8) |
|  | 2029 | 165 (8.0) |
| Competency to be assessed | Comprehensive clinical performance | 1,097 (53.3) |
|  | Clinical skills | 493 (24) |
|  | Writing medical records | 207 (10.1) |
|  | Communication | 82 (4) |
|  | Identifying cases to transfer | 179 (8.7) |

Table 1-2-3. Number of “required” responses for top 10 clinical presentation among those who stated that the CSE is essential (N = 2,058)

| **Rank** | **Symptom** | **n** | **Share of total respondents** |
| --- | --- | --- | --- |
| **1** | Headache | 1690 | 82.1% |
| **2** | Backache | 1683 | 81.8% |
| **3** | Chronic abdominal pain/dyspepsia/heartburn | 1628 | 79.1% |
| **4** | Dizziness | 1601 | 77.8% |
| **5** | numbness/dysesthesia | 1586 | 77.1% |
| **6** | Neck pain | 1550 | 75.3% |
| **7** | Arthralgia/arthrocele | 1535 | 74.6% |
| **8** | Diarrhea | 1532 | 74.4% |
| **9** | Acute stomachache | 1524 | 74.1% |
| **10** | Dysmenorrhea | 1516 | 73.7% |

**Abbreviations.** CSE = clinical skill examination

Table 1-2-4. Number of “required” responses for top 10 basic clinical skill among those who stated that the CSE is essential (N = 2,058)

| No | Basic clinical skill | n | Share of total respondents |
| --- | --- | --- | --- |
| 1 | Acupuncture | 1689 | 82.10% |
| 2 | Abdominal diagnosis (in OM) | 1677 | 81.50% |
| 3 | Pharmacopuncture | 1580 | 76.80% |
| 4 | Cupping | 1536 | 74.60% |
| 5 | Motor system examination | 1518 | 73.80% |
| 6 | Chuna | 1516 | 73.70% |
| 7 | Medical records/medical certificate | 1446 | 70.30% |
| 8 | Moxibustion | 1419 | 69.00% |
| 9 | Tongue diagnosis | 1406 | 68.30% |
| 10 | Pulse examination | 1370 | 66.60% |

**Abbreviations**. CSE = clinical skill examination; OM = Oriental medicine.

*1-3. Advisory board meeting with the participation of the Association of Korean Medicine Colleges*

During August 2022, advisory board meeting with the participation of the Association of Korean Medicine Colleges was held. At this meeting, the results of an email-based survey of oriental medicine doctors were shared (i.e., ‘1-2. Email-based survey of oriental medicine doctors’). Afterwards, deans of oriental medicine schools expressed their views on the introduction of the clinical skills examination into the Korean Oriental Medicine Licensing Examination. The following opinions were suggested and reflected in the progress of the Delphi round: (1) Various opinions must be gathered from various experts.; (2) A model that can be implemented must be established.; and (3) The current status of oriental medicine schools should be reflected.

*1-4. Email-based survey of professors at 12 oriental medicine schools*

During September 2022, professors at 12 oriental medicine schools were asked to respond not only to the necessity and implementation strategies of the clinical skills examination, but also to respond about their willingness and awareness to participate in the operation of the clinical skills examination when it is introduced into the Korean Oriental Medicine Licensing Examination.

Table 1-4-1. Summary of survey results for professors at the 12 schools of OM (N=206)

| Question | Response |  |  | n | Share of total respondents (%) |
| --- | --- | --- | --- | --- | --- |
| Necessity | Strongly positive |  |  | 111 | 53.9 |
|  | Slightly positive |  |  | 79 | 38.3 |
|  | Neutral |  |  | 13 | 6.3 |
|  | Slightly negative |  |  | 2 | 1 |
|  | Strongly negative |  |  | 1 | 0.5 |
| Year of Introduction of CSE | 2025 |  |  | 78 | 37.9 |
|  | 2026 |  |  | 30 | 14.6 |
|  | 2027 |  |  | 43 | 20.9 |
|  | 2028 |  |  | 16 | 7.5 |
|  | 2029 |  |  | 32 | 15.5 |
|  | Others |  |  | 7 | 3.4 |
| Supervising and implementing the organization of CSE | KHPLEI | | | 142 | 68.9 |
|  | Regional autonomy under the responsibility of KHPLEI | | | 55 | 26.7 |
|  | KHPLEI and University | | | 8 | 3.9 |
|  | University | | | 1 | 0.5 |
| Composition of CSE | 9 CPXs and 1 combined-OSCE of 3 skills (the CSE model of MD in South Korea, 2021~current) | | | 79 | 38.3 |
|  | 3 result-assessments and 3 procedure-assessments (the CSE model of dentists in South Korea) | | | 72 | 35 |
|  | 6 CPXs, 6 inter-station tests, and 6 single OSCEs (the CSE model of MD in South Korea, 2009~2020) | | | 29 | 14.1 |
|  | Case analysis, 4 inter-student demonstrations (similar to OSCE), and 2 oral tests (the CSE model of TCM doctor in China) | | | 12 | 5.8 |
|  | 12 CPXs and 12 patient notes (the CSE model of MD in US (i.e., USMLE)) | | | 10 | 4.9 |
|  | Other | | | 4 | 2.2 |
| Test time per station | 15 minutes | | | 79 | 38.3 |
|  | 10 minutes | | | 70 | 34 |
|  | 12 minutes | | | 52 | 25.2 |
|  | Other (20 minutes, 20~30 minutes) | | | 5 | 2.4 |
| Total test time of CSE | 3 hours | | | 108 | 52.4 |
|  | 2 hours. | | | 82 | 39.8 |
|  | 4 hours. | | | 7 | 3.4 |
|  | 6 hours. | | | 4 | 1.9 |
|  | Other (1 hour, 150 minutes) | | | 4 | 1.9 |
|  | 5 hours. | | | 1 | 0.5 |
| Timing and eligibility for CSE | After the written test, only prospective graduates who have passed the written test. | | | 94 | 45.6 |
|  | Before the written test, prospective graduate. | | | 51 | 24.8 |
|  | After the written test, prospective graduate. | | | 37 | 18 |
|  | Regardless of the written test and eligibility. | | | 24 | 11.7 |
| Determination of pass | Passing both the written and clinical skills exams, respectively. | | | 147 | 71.4 |
|  | Passing according to the combined score of the written test and clinical skills exams. | | | 56 | 27.2 |
|  | Other | | | 3 | 1.5 |
| Rating HT, PE, and ED of CPX  (PPI is scored by 1 SP who is acting) | By 1 SP who is observing |  |  | 98 | 47.6 |
|  | By 1 professor |  |  | 56 | 27.2 |
|  | By 2 professors |  |  | 44 | 21.4 |
|  | By 1 SP who is acting |  |  | 5 | 2.4 |
|  | Other |  |  | 3 | 1.5 |
| Rating OSCE | By 1 university teacher at schools of OM (scoring) |  |  | 135 | 65.5 |
|  | By 2 university teachers at schools of OM |  |  | 71 | 34.5 |
| Sex | Male |  |  | 152 | 73.8 |
|  | Female |  |  | 54 | 26.2 |
| Period | <5 years |  |  | 49 | 23.8 |
|  | 5–10 years |  |  | 35 | 17 |
|  | 10–15 years |  |  | 39 | 18.9 |
|  | 15–20 years |  |  | 33 | 16 |
|  | ≥20 years |  |  | 50 | 24.3 |
| Position | Full-time lecturer |  |  | 12 | 5.8 |
|  | Assistant professor |  |  | 46 | 22.3 |
|  | Associate professor |  |  | 47 | 22.8 |
|  | Professor |  |  | 100 | 48.5 |
|  | Other |  |  | 1 | 0.5 |
| Major | Basic OM |  |  | 60 | 29.13 |
|  | Clinical OM |  |  | 144 | 69.9 |
|  | Other |  |  | 2 | 0.97 |
| Affiliation | Ga Chon University |  |  | 14 | 6.8 |
|  | Kyung Hee University |  |  | 18 | 8.7 |
|  | Deagu Haany University |  |  | 30 | 14.6 |
|  | Deajeon University |  |  | 25 | 12.1 |
|  | Dong Guk University |  |  | 13 | 6.3 |
|  | Dong Shin University |  |  | 8 | 3.9 |
|  | Dong Eui University |  |  | 21 | 10.2 |
|  | Pusan National University |  |  | 24 | 11.7 |
|  | Sang Ji University |  |  | 13 | 6.3 |
|  | Se Myung University |  |  | 5 | 2.4 |
|  | Woo Suk University |  |  | 10 | 4.9 |
|  | Won Kwang University |  |  | 25 | 12.1 |

**Abbreviations**. CSE = clinical skill examination; CPX = clinical practice examination; ED = education; HT = history taking; KHPLEI = Korea Health Personnel Licensing Examination Institute; OM = Oriental medicine; OSCE = objective structured clinical examination; PE = physical examination; PPI = patient-physician interaction; SP = standardized patient.

**Supplement 2. Protocol of the study**

After the setting of the problem area (**Supplement 1**), the Delphi method was implemented over three rounds. Each round was conducted as an email-based survey to a panel of experts. Anonymity was guaranteed, and controlled feedback was provided. The objectives of the first, second, and third rounds were distinguished.

*2-1. First round*

In the first round (9/15/2022 to 9/22/2022), opinions were gathered from the expert panel on the list of potential candidates for the CPX and OSCE. The list included clinical presentations as part of the CPX and basic clinical skills as part of the OSCE, obtained from the oriental medicine doctor survey (i.e., 20% or greater acceptance rate for the necessity) during the setting of the problem area process (**Supplementary 4**). The validity of introducing each item into the NE for oriental medicine doctors was measured with six (for basic clinical skills) or seven (for clinical presentations) questions, including “necessity,” “feasibility,” and RUMBA (i.e., “relevant,” “understandable,” “measurable,” “behavioral,” and “achievable”). The questions were to be answered on a 3- or 5-point Likert scale. As the OSCE list was confirmed as implementable in the course of our comprehensive literature review, the feasibility question was excluded. Open-ended questions enabled the free expression of opinions vis-à-vis clinical presentations or basic clinical skills. To discuss comments raised in the open-ended questions, four subcommittees were formed by requesting official recommendations from related official organizations with regard to pulse examination, acupuncture, Chuna, and Sasang constitution. One or two online meetings of the subcommittees were held and chaired by the authors.

*2-2. Second and third rounds*

In the second (9/27/2022 to 10/6/2022) and third (10/22/2022 to 11/3/2022) rounds, the validity of detailed methods for the CSE was investigated. The questions were based on the results of the oriental medicine doctor survey during the process of the setting of the problem area, and the agreement of the expert panel on the most frequent options of professors at the 12 oriental medicine schools was evaluated as “yes” or “no.” The third round included questions on which no consensus was reached in the second round. In this round, options other than the most frequent one according to the professor’s response could also be selected. The results of discussions in the subcommittees were provided, and the expert panel was asked to respond whether or not it agreed with the results of the discussions. The degree of agreement on the results of the discussions by subcommittees was classified into “relevant” and “measurable” (5-point Likert scale).
