# Supplementary material for: Implementation strategy for introducing a clinical skills examination to the Korean Oriental Medicine Licensing Examination: a mixed-method modified Delphi study
Source: J Educ Eval Health Prof. 2023 Jul 17;20:23. doi: 10.3352/jeehp.2023.20.23 (PMC10432826; doi:10.3352/jeehp.2023.20.23)
Supplement: Supplementary file 2 — Supplement 2. Protocol of the study. [file jeehp-20-23-suppl2.docx]

**Supplement 2. Protocol of the study**

To set the problem area, the authors conducted four investigations for clarification: a comprehensive literature review, an email-based survey of OMDs registered with the Association of Korean Medicine (response rate: 9.28% (2,221/23,946) (**Supplementary 2**) and of professors at 12 OM schools (response rate: 41.4%) (206/500) (**Supplementary 3**), and an advisory board meeting with the participation of the Association of Korean Medicine Colleges. The Delphi method was implemented over three rounds. Each round was conducted as an email-based survey to a panel of experts. Anonymity was guaranteed, and controlled feedback was provided. The objectives of the first, second, and third rounds were distinguished.

*First round*

In the first round (9/15/2022 to 9/22/2022), opinions were gathered from the expert panel on the list of potential candidates for the CPX and OSCE. The list included 87 clinical presentations as part of the CPX and 31 basic clinical skills as part of the OSCE, obtained from the OMD survey (i.e., 20% or greater acceptance rate for the necessity) during the setting of the problem area process (**Supplementary 4**). The validity of introducing each item into the NE for OMDs was measured with six (for basic clinical skills) or seven (for clinical presentations) questions, including “necessity,” “feasibility,” and RUMBA (i.e., “relevant,” “understandable,” “measurable,” “behavioral,” and “achievable”). The questions were to be answered on a 3- or 5-point Likert scale. As the OSCE list was confirmed as implementable in the course of our comprehensive literature review, the feasibility question was excluded. Open-ended questions enabled the free expression of opinions vis-à-vis clinical presentations or basic clinical skills. To discuss comments raised in the open-ended questions, four subcommittees were formed by requesting official recommendations from related official organizations with regard to pulse examination, acupuncture, Chuna, and Sasang constitution. One or two online meetings of the subcommittees were held and chaired by the authors.

*Second and third rounds*

In the second (9/27/2022 to 10/6/2022) and third (10/22/2022 to 11/3/2022) rounds, the validity of detailed methods for the CSE was investigated. The questions were based on the results of the OMD survey during the process of the setting of the problem area, and the agreement of the expert panel on the most frequent options of professors at the 12 OM schools was evaluated as “yes” or “no.” The third round included questions on which no consensus was reached in the second round. In this round, options other than the most frequent one according to the professor’s response could also be selected. The results of discussions in the subcommittees were provided, and the expert panel was asked to respond whether or not it agreed with the results of the discussions. The degree of agreement on the results of the discussions by subcommittees was classified into “relevant” and “measurable” (5-point Likert scale).

**Supplement 2. Summary of survey results for Oriental medicine doctors**

**2-1. Demographic data of the respondents (N = 2,221)**

| Variables | | N | Share of total respondents (%) |
| --- | --- | --- | --- |
| Age (years) | 20s | 294 | 13.2 |
|  | 30s | 893 | 40.2 |
|  | 40s | 598 | 26.9 |
|  | 50s | 350 | 15.8 |
|  | ≥60s | 86 | 3.9 |
| Sex | Male | 1,555 | 70 |
|  | Female | 666 | 30 |
| Affiliation | University (basic department) | 28 | 1.3 |
|  | University (clinical department) | 59 | 2.7 |
|  | Oriental Medicine Hospital | 454 | 20.4 |
|  | Oriental Medicine Clinic | 1,372 | 61.8 |
|  | Public Medical Institution | 221 | 10 |
|  | Other (Research Institution) | 87 | 3.9 |
| Resident education | None | 1,528 | 68.8 |
|  | Completed Internship only | 143 | 6.4 |
|  | Complete Residency | 550 | 24.8 |
| License acquisition year | 2018–2022 | 640 | 28.8 |
|  | 2013–2017 | 474 | 21.3 |
|  | 2008–2012 | 335 | 15.1 |
|  | 2003–2007 | 278 | 12.5 |
|  | 1998–2002 | 238 | 10.7 |
|  | 1993–1997 | 110 | 5 |
|  | 1988–1992 | 91 | 4.1 |
|  | 1983–1987 | 33 | 1.5 |
|  | before 1982 | 22 | 1 |

**2-2. Summary of survey results (N = 2,221)**

| Questions | Responses | n (Share of total respondents (%)) |
| --- | --- | --- |
| Necessity | Strongly positive | 1,060 (47.7) |
|  | Slightly positive | 759 (34.2) |
|  | Neutral | 239 (10.8) |
|  | Slightly negative | 82 (3.7) |
|  | Strongly negative | 81 (3.6) |
| Year of introduction of clinical skills examination | 2025 | 1,328 (64.5) |
|  | 2026 | 221 (10.7) |
|  | 2027 | 245 (11.9) |
|  | 2028 | 99 (4.8) |
|  | 2029 | 165 (8.0) |
| Competency to be assessed | Comprehensive clinical performance | 1,097 (53.3) |
|  | Clinical skills | 493 (24) |
|  | Writing medical records | 207 (10.1) |
|  | Communication | 82 (4) |
|  | Identifying cases to transfer | 179 (8.7) |

**2-3. Number of “required” responses for top 20 clinical presentation among those who stated that the CSE is essential (N = 2,058)**

| **Rank** | **Symptom** | **n** | **Share of total respondents** |  | **Rank** | **Symptom** | **n** | **Share of total respondents** |
| --- | --- | --- | --- | --- | --- | --- | --- | --- |
| 1 | Headache | 1690 | 82.1% |  | 11 | Omalgia | 1510 | 73.4% |
| 2 | Backache | 1683 | 81.8% |  | 12 | Somnipathy | 1500 | 72.9% |
| 3 | Chronic abdominal pain/dyspepsia/heartburn | 1628 | 79.1% |  | 13 | Cough | 1495 | 72.6% |
| 4 | Dizziness | 1601 | 77.8% |  | 14 | Muscular weakness | 1475 | 71.7% |
| 5 | numbness/dysesthesia | 1586 | 77.1% |  | 15 | Nausea/ vomit | 1469 | 71.4% |
| 6 | Neck pain | 1550 | 75.3% |  | 16 | Constipation | 1458 | 70.8% |
| 7 | Arthralgia/arthrocele | 1535 | 74.6% |  | 17 | Knee pain | 1454 | 70.7% |
| 8 | Diarrhea | 1532 | 74.4% |  | 18 | Fever | 1449 | 70.4% |
| 9 | Acute stomachache | 1524 | 74.1% |  | 19 | Chest pain/epigastric displeasure | 1438 | 69.9% |
| 10 | Dysmenorrhea | 1516 | 73.7% |  | 20 | Fatigue | 1427 | 69.3% |

**Abbreviations.** CSE = clinical skill examination

**2-4. Number of “required” responses for top 20 basic clinical skill among those who stated that the CSE is essential (N = 2,058)**

| No | Basic clinical skill | n | Share of total respondents |
| --- | --- | --- | --- |
| 1 | Acupuncture | 1689 | 82.10% |
| 2 | Abdominal diagnosis (in OM) | 1677 | 81.50% |
| 3 | Pharmacopuncture | 1580 | 76.80% |
| 4 | Cupping | 1536 | 74.60% |
| 5 | Motor system examination | 1518 | 73.80% |
| 6 | Chuna | 1516 | 73.70% |
| 7 | Medical records/medical certificate | 1446 | 70.30% |
| 8 | Moxibustion | 1419 | 69.00% |
| 9 | Tongue diagnosis | 1406 | 68.30% |
| 10 | Pulse examination | 1370 | 66.60% |
| 11 | Sensory system examination | 1335 | 64.90% |
| 12 | Reflex test | 1295 | 62.90% |
| 13 | Wound dressing | 1110 | 53.90% |
| 14 | Measurement of blood pressure | 1109 | 53.90% |
| 15 | Cardiopulmonary resuscitation/defibrillation | 1068 | 51.90% |
| 16 | Lung examination | 1035 | 50.30% |
| 17 | Thread embedding therapy | 942 | 45.80% |
| 18 | Chest X-ray presentation | 940 | 45.70% |
| 19 | Abdominal diagnosis | 926 | 45.00% |
| 20 | Cranial nerve examination | 868 | 42.20% |

**Abbreviations**. CSE = clinical skill examination; OM = Oriental medicine.

**Supplement 3. Summary of survey results for professors at the 12 schools of OM (N=206)**

| Question | Response |  |  | n | Share of total respondents (%) |
| --- | --- | --- | --- | --- | --- |
| Necessity | Strongly positive |  |  | 111 | 53.9 |
|  | Slightly positive |  |  | 79 | 38.3 |
|  | Neutral |  |  | 13 | 6.3 |
|  | Slightly negative |  |  | 2 | 1 |
|  | Strongly negative |  |  | 1 | 0.5 |
| Year of Introduction of CSE | 2025 |  |  | 78 | 37.9 |
|  | 2026 |  |  | 30 | 14.6 |
|  | 2027 |  |  | 43 | 20.9 |
|  | 2028 |  |  | 16 | 7.5 |
|  | 2029 |  |  | 32 | 15.5 |
|  | Others |  |  | 7 | 3.4 |
| Supervising and implementing the organization of CSE | KHPLEI | | | 142 | 68.9 |
|  | Regional autonomy under the responsibility of KHPLEI | | | 55 | 26.7 |
|  | KHPLEI and University | | | 8 | 3.9 |
|  | University | | | 1 | 0.5 |
| Composition of CSE | 9 CPXs and 1 combined-OSCE of 3 skills (the CSE model of MD in South Korea, 2021~current) | | | 79 | 38.3 |
|  | 3 result-assessments and 3 procedure-assessments (the CSE model of dentists in South Korea) | | | 72 | 35 |
|  | 6 CPXs, 6 inter-station tests, and 6 single OSCEs (the CSE model of MD in South Korea, 2009~2020) | | | 29 | 14.1 |
|  | Case analysis, 4 inter-student demonstrations (similar to OSCE), and 2 oral tests (the CSE model of TCM doctor in China) | | | 12 | 5.8 |
|  | 12 CPXs and 12 patient notes (the CSE model of MD in US (i.e., USMLE)) | | | 10 | 4.9 |
|  | Other | | | 4 | 2.2 |
| Test time per station | 15 minutes | | | 79 | 38.3 |
|  | 10 minutes | | | 70 | 34 |
|  | 12 minutes | | | 52 | 25.2 |
|  | Other (20 minutes, 20~30 minutes) | | | 5 | 2.4 |
| Total test time of CSE | 3 hours | | | 108 | 52.4 |
|  | 2 hours. | | | 82 | 39.8 |
|  | 4 hours. | | | 7 | 3.4 |
|  | 6 hours. | | | 4 | 1.9 |
|  | Other (1 hour, 150 minutes) | | | 4 | 1.9 |
|  | 5 hours. | | | 1 | 0.5 |
| Timing and eligibility for CSE | After the written test, only prospective graduates who have passed the written test. | | | 94 | 45.6 |
|  | Before the written test, prospective graduate. | | | 51 | 24.8 |
|  | After the written test, prospective graduate. | | | 37 | 18 |
|  | Regardless of the written test and eligibility. | | | 24 | 11.7 |
| Determination of pass | Passing both the written and clinical skills exams, respectively. | | | 147 | 71.4 |
|  | Passing according to the combined score of the written test and clinical skills exams. | | | 56 | 27.2 |
|  | Other | | | 3 | 1.5 |
| Rating HT, PE, and ED of CPX  (PPI is scored by 1 SP who is acting) | By 1 SP who is observing |  |  | 98 | 47.6 |
|  | By 1 professor |  |  | 56 | 27.2 |
|  | By 2 professors |  |  | 44 | 21.4 |
|  | By 1 SP who is acting |  |  | 5 | 2.4 |
|  | Other |  |  | 3 | 1.5 |
| Rating OSCE | By 1 university teacher at schools of OM (scoring) |  |  | 135 | 65.5 |
|  | By 2 university teachers at schools of OM |  |  | 71 | 34.5 |
| Sex | Male |  |  | 152 | 73.8 |
|  | Female |  |  | 54 | 26.2 |
| Period | <5 years |  |  | 49 | 23.8 |
|  | 5–10 years |  |  | 35 | 17 |
|  | 10–15 years |  |  | 39 | 18.9 |
|  | 15–20 years |  |  | 33 | 16 |
|  | ≥20 years |  |  | 50 | 24.3 |
| Position | Full-time lecturer |  |  | 12 | 5.8 |
|  | Assistant professor |  |  | 46 | 22.3 |
|  | Associate professor |  |  | 47 | 22.8 |
|  | Professor |  |  | 100 | 48.5 |
|  | Other |  |  | 1 | 0.5 |
| Major | Basic OM |  |  | 60 | 29.13 |
|  | Clinical OM |  |  | 144 | 69.9 |
|  | Other |  |  | 2 | 0.97 |
| Affiliation | Ga Chon University |  |  | 14 | 6.8 |
|  | Kyung Hee University |  |  | 18 | 8.7 |
|  | Deagu Haany University |  |  | 30 | 14.6 |
|  | Deajeon University |  |  | 25 | 12.1 |
|  | Dong Guk University |  |  | 13 | 6.3 |
|  | Dong Shin University |  |  | 8 | 3.9 |
|  | Dong Eui University |  |  | 21 | 10.2 |
|  | Pusan National University |  |  | 24 | 11.7 |
|  | Sang Ji University |  |  | 13 | 6.3 |
|  | Se Myung University |  |  | 5 | 2.4 |
|  | Woo Suk University |  |  | 10 | 4.9 |
|  | Won Kwang University |  |  | 25 | 12.1 |

**Abbreviations**. CSE = clinical skill examination; CPX = clinical practice examination; ED = education; HT = history taking; KHPLEI = Korea Health Personnel Licensing Examination Institute; OM = Oriental medicine; OSCE = objective structured clinical examination; PE = physical examination; PPI = patient-physician interaction; SP = standardized patient.
